# Soft tissue artifact evaluation of the cervical spine in motion patterns of flexion and lateral bending: a preliminary study

**DOI:** 10.7717/peerj.1893

**Published:** 2016-03-31

**Authors:** Jiajia Wang, Zhongwen Lui, Zhihui Qian, Luquan Ren

**Affiliations:** 1 College of Agricultural Engineering, Henan University of Science and Technology, Luo Yang He Nan, China; 2 Key Laboratory of Bionic Engineering (Ministry of Education, China), Jilin University, Chang Chun JiLin, China; 3 Radiology Department, China-Japan Union Hospital of Jilin University, Chang Chun JiLin, China

**Keywords:** Cervical spine, Soft tissue artifact, Magnetic resonance imaging

## Abstract

**Background.** Soft tissue artifact (STA) is increasingly becoming a focus of research as the skin marker method is widely employed in motion capture technique. At present, medical imaging methods provide reliable ways to investigate the cervical STA. Among these approaches, magnetic resonance imaging (MRI) is a highly preferred tool because of its low radiation.

**Methods.** In the study, the 3D spatial location of vertebral landmarks and corresponding skin markers of the spinous processes of the second (C2), fifth (C5), and sixth (C6) cervical levels during flexion and lateral bending were investigated. A series of static postures were scanned using MRI. Skin deformation was obtained by the Mimics software.

**Results.** Results shows that during flexion, the maximum skin deformation occurs at C6, in the superior–inferior (*Z*) direction. Upon lateral bending, the maximum skin displacement occurs at C2 level, in the left–right (*Y*) direction. The result presents variability of soft tissue in the terms of direction and magnitude, which is consistent with the prevailing opinion.

**Discussion.** The results testified variability of cervical STA. Future studies involving large ranges of subject classification, such as age, sex, height, gravity, and etc. should be performed to completely verify the existing hypothesis on human cervical skin deformation.

## Introduction

The skin marker method has been widely applied to investigate cervical kinematics. This approach provides a non-invasive means to understand normal and abnormal cervical motion. Soft tissue artifacts (STA) has been regarded as the most critical source of error in human movement analysis (Leardini et al., 2005); it has been widely investigated in the lower limb (Akbarshahi et al., 2010; Gao & Zheng, 2008; Ryu, Choi & Chung, 2009) and upper limb (Cutti et al., 2005).

Various invasive techniques have been used to investigate the STA of human body parts, including intracortical pins (Reinschmidt et al., 1997), external fixators (Cappozzo et al., 1996), and percutaneous trackers (Manal et al., 2000). These invasive methods are suitable for patients who require surgery and those who volunteer as research subjects. Medical techniques have been widely used in cervical disease diagnosis and motion analysis, as well as in the validation of surface marker placement accuracy (Gadotti & Magee, 2013). Compared with Roentgen photogrammetry (Tashman & Anderst, 2002) and video fluoroscopy (Wu et al., 2007), magnetic resonance imaging (MRI) (Ishii et al., 2006; Nagamoto et al., 2011) has the advantage of involving less radiation. In recent decades, MRI application has expanded from clinical imaging to biomechanical kinematic analyses because of the technique’s low radiation (Karhu et al., 1999). Although the kinematic characteristics of the spinal system are complex, STA investigation during spinal motion has been conducted previously and reported as follows.

Mörl & Blickhan (2006) revealed the linear relationship between the 3D movement of skin markers and the corresponding lumbar vertebrae in different postures using open MRI. Hashemirad et al. (2013) confirmed the validity and reliability of the surface skin marker method aided with fluoroscopy in measuring intersegmental mobility at L2–L3 and L3–L4 during lateral bending. For the cervical spine, Wu et al. (2007) explored the rotational angles of C2, C3, C5, and C7 levels by skin marker method aided with fluoroscopy during flexion/extension and lateral bending. The study demonstrated the feasibility of the skin marker method in obtaining cervical segmental rotational angles. Moreover, MRI technology is a desirable method because it allows the non-invasive 3D imaging of true joint bony structures and the surrounding soft tissues (Sangeux et al., 2006).

In this study, the cervical skin movement of young normal adults in flexion and lateral bending is preliminarily measured by the 3D MRI technique. Spatial displacement of skin markers and corresponding vertebral landmarks are obtained, including the direction and magnitude of relative movement.

## Materials and Methods

Three male volunteers participated in the study. The mean (standard deviation) age, body mass, and height were 24 (1) years old, 64 (2) kg, and 170 (5) cm, respectively. All subjects provided informed consent prior to testing and have no history of cervical trauma, bone pathology, and arthritic or other inflammatory disorders. All the study designs and consent forms were approved by the Institutional Ethical Review Board Committee of Jilin University, Changchun, China (No. 20130629).

Three hemisphere-shaped MRI markers with a diameter of 5 mm were attached to the nape of each subject. Each marker was applied to mark the spinous processes of the second (C2), fifth (C5), and sixth cervical vertebrae (C6), as palpated in a flexed posture.

**Figure 1:**
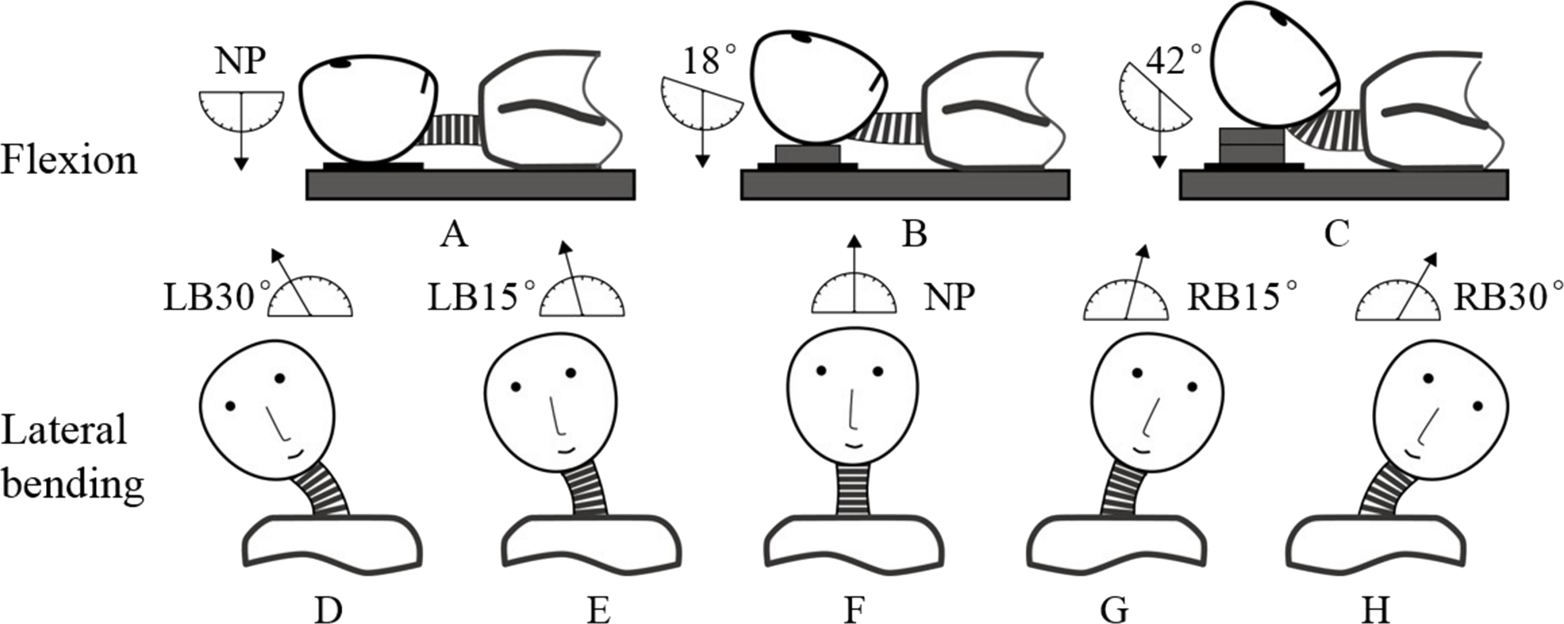
Three postures in flexion: (A) neutral position (NP), (B) first flexion at 18°, and (C) second flexion at 42°. Five postures in lateral bending: (D) left bending at 30°(LB30°), (E) left bending at 15°(LB15°), (F) neutral position (NP), (G) right bending at 15°(RB15°), and (H) right bending at 30°(RB30°).

Each subject was instructed to lie down on his back and then assume different postures during scanning, including 3 positions in flexion and 5 positions in lateral bending (Fig. 1). In this work, the maximum ranges of motion in flexion and lateral bending lied within the average ranges of the young people as reported by Jaejin & Myung (2015). The motion angles approximated to the intermediate positions during each motion patterns were selected as follows, respectively.

The neutral position (Fig. 1A) and two other positions in which the neck was flexed at angles 18°(Fig. 1B) and 42°(Fig. 1C) were determined using a protractor with respect to a vertically hanging object. The positions were fixed by placing polyurethane plates under the subject’s head. The neutral position (Fig. 1F) and four other positions in which the neck was extended at different angles were determined by a round-shaped board with 5°scales. The four positions were as follows: left bending at 15°(LB15°; Fig. 1E), left bending at 30°(LB30°; Fig. 1D), right bending at 15°(RB15°; Fig. 1G), and right bending at 30°(RB30°; Fig. 1H).

**Figure 2:**
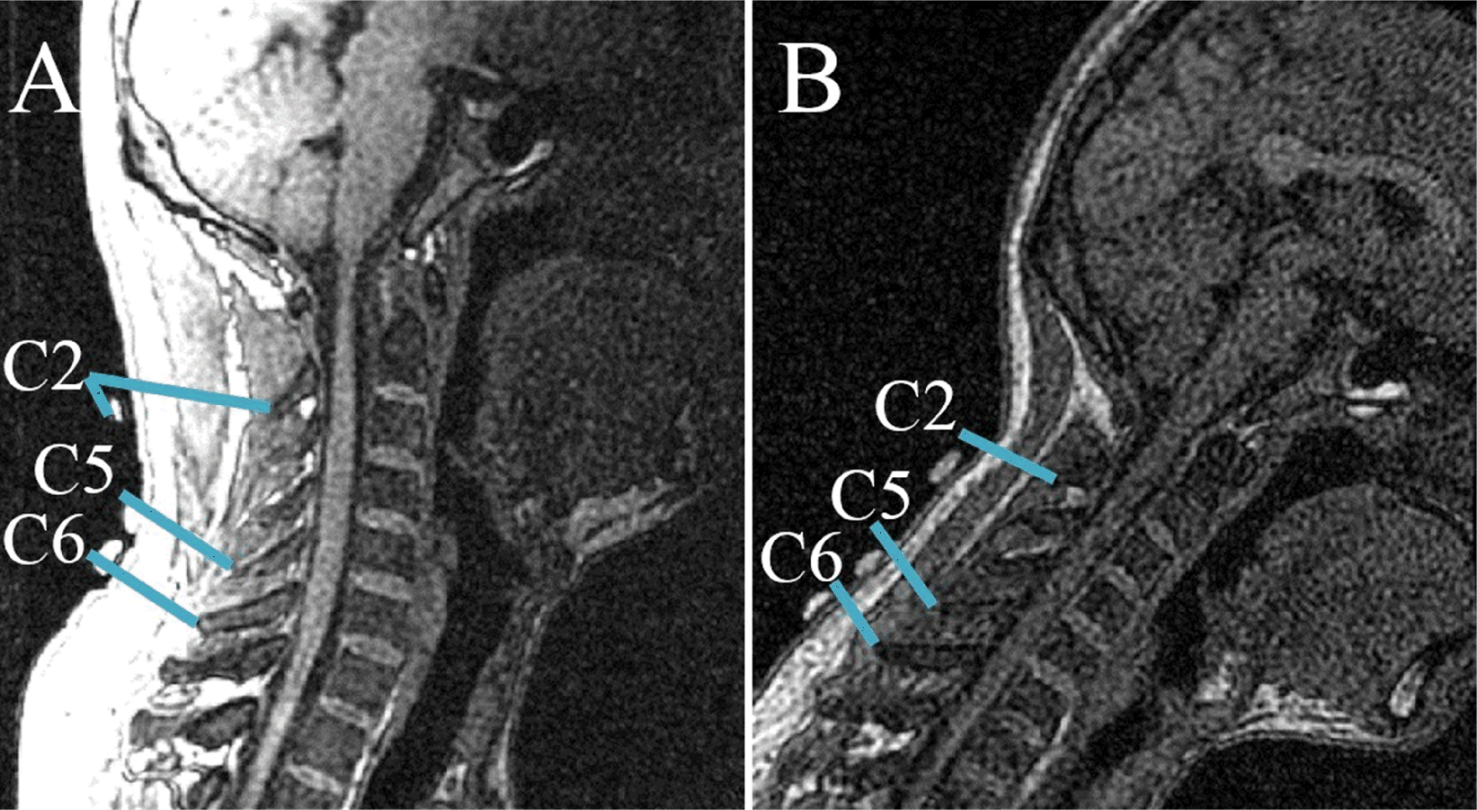
Relative positions of skin markers and corresponding spinous processes/vertebral landmarks in the sagittal plane at two postures: (A) NP; (B) second flexion position. The ends of short blue lines point to the skin markers and corresponding vertebral landmarks, respectively.

The two series of cervical postures, including flexion and lateral bending, were imaged using a 1.5T commercial MR system (Signa CV, General Electric, United Kingdom) along with a torso phased array coil. A 3D fast gradient recalled acquisition in the steady state pulse sequence was used with repetition time/echo time of 3.9 ms/1.8 ms. The slice thickness was 1.8 mm with no interslice gap. The flip angle was 15°with a 24 cm field-of-view and a 256 × 224 in-plane acquisition matrix. Imaging of one position lasted for approximately 3.5 min. The acquisition data were saved in Dicom format and processed by the Mimics software (V10.01; Materialise, Leuven Belgium). Each skin marker was then digitized using the point at the marker center. Meanwhile, the positional information of the tip of each spinous process was digitized in three anatomic planes. The position relationships between skin markers and corresponding spinous processes were clearly shown in the sagittal plane, the ends of short blue lines were pointed at the skin markers and corresponding vertebral landmarks, respectively (Fig. 2). The global reference frame was defined as follows. The +*Z* axis pointed upward and positioned parallel to the field of gravity, whereas the +*X* and +*Y* axes lied in a plane perpendicular to the *Z* axis and pointed toward the anterior and left lateral directions, respectively.

The special positions of each spinous process and corresponding skin surface marker at each observed posture were measured. The following equations were used to determine the spatial 3D (*X*−*i*, *Y*−*j*, *Z*−*k*) skin displacement of each cervical level at each different posture:

(1) \begin{eqnarray*}{S}^{i,j,k}=Motio{n}_{vertebral-landmark}^{i,j,k}-Motio{n}_{skin-marker}^{i,j,k}\cdot \end{eqnarray*}

The skin displacements were calculated by subtracting the displacement of the skin marker from the corresponding displacement of the vertebral landmarks. In the abovementioned equations, $Motio{n}_{vertebral-landmark}^{i}$, $Motio{n}_{vertebral-landmark}^{j}$, and $Motio{n}_{vertebral-landmark}^{k}$ represents the vertebral landmark displacements in the *X*, *Y*, and *Z* directions, respectively. Similarly, $Motio{n}_{skin-marker}^{i}$, $Motio{n}_{skin-marker}^{j}$, and $Motio{n}_{skin-marker}^{k}$ represents the skin marker displacements in the *X*, *Y*, and *Z* directions, respectively. The absolute value of *S*^*i*,*j*,*k*^ denotes the amplitude of the skin movement. The sign of *S*^*i*,*j*,*k*^ represents the direction of the relative movement between vertebral landmarks and the corresponding skin markers. In particular, +*S*^*i*^ implies that the vertebral landmarks moved relative to the corresponding skin markers along the +*X* direction.

## Results

The skin deformation of three cervical levels during flexion and lateral bending were investigated, and the results are shown in Tables 1 and 2. The tables display the amplitude and directional information of cervical skin deformation, from which the linear relationship between the motion of vertebral landmarks and the corresponding skin markers was deduced.

**Table 1:** Skin displacement of the three subjects (S1, S2, and S3) at two postures (first and second flexion) in flexion.

Rotations	Vertebrae	*S* (mm)									Range of |*S*| (mm)		
		*X*			*Y*			*Z*			*X*	*Y*	*Z*
		S1	S2	S3	S1	S2	S3	S1	S2	S3	–	–	–
First flexion	C2	−0.5	−0.5	−1.5	7.2	1.5	0	0	−2.5	−1	0.5–1.5	0–7.2	0–2.5
	C5	−5	−2	0.5	0	4.5	−1.5	2.5	1.5	−4.5	0.5–5	0–4.5	1.5–4.5
18°	C6	−2	4	−1.5	0	0	−4.5	2	3.5	−9.5	2–4	0–4.5	2–9.5
Second flexion	C2	−8.5	−11.5	0	7.2	0	13.5	−3	−5.5	1	0–11.5	0–13.5	1–5.5
	C5	−6.5	12.5	2	0	7.5	−1.5	2.5	8	−5	2–12.5	0–7.5	2.5–8
42°	C6	−5	0.5	−0.5	3.6	1.5	−3	4.5	9	−13.5	0.5–5	1.5–3.6	4.5–13.5

**Table 2:** Skin displacement of the three subjects at four postures (LB30°, LB15°, RB15°, and RB30°) in lateral bending.

Rotations	Vertebrae	*S* (mm)									Range of |*S*| (mm)		
		*X*			*Y*			*Z*			*X*	*Y*	*Z*
		S1	S2	S3	S1	S2	S3	S1	S2	S3	–	–	–
	C2	3	14.5	4.5	−20.5	3.3	−17.5	7.5	7	2	3–14.5	3.3–20.5	2–7.5
LB30°	C5	0	6.6	16.5	−0.5	5.4	−3.5	0.5	3.5	−6.5	0–16.5	0.5–5.4	0.5–6.5
	C6	−1.5	7	12	1.5	6.6	1	1	5	−8.5	7–12	1–6.6	1–8.5
	C2	0	10.9	3	−20	0.8	−16	1	5	2	0–10.9	0.8–20	1–5
LB15°	C5	−3	7.4	21	5.5	2.9	−7	2	1.5	−6.5	3–21	2.9–7	2–6.5
	C6	0	5.2	16.5	3.5	4.6	−3.5	4	4	−7.5	0–16.5	3.5–4.6	4–7.5
	C2	1.5	12.7	1.5	−4	4.3	5	0.5	4.8	10.5	1.5–12.7	4–5	0.5–10.5
RB15°	C5	3	6.6	16.5	10.5	9.4	−3.5	−1.5	1.5	−6.5	3–16.5	3.5–10.5	1.5–6.5
	C6	1.5	8.8	15	9.5	7.1	−1.5	2	3.8	−8	1.5–15	1.5–9.5	2–8
	C2	3	14.5	4.5	−3.5	5.3	2	−1	8.3	16	3–14.5	2–5.3	1–16
RB30°	C5	1.5	7.4	13.5	11	8.4	−19.5	−1	4.5	−13	1.5–13.5	8.4–19.5	1–13
	C6	1.5	7	15	9.5	6.6	−13	2	4.3	−8	1.5–15	6.6–13	2–8

### Amplitude and directional analysis of skin deformation

Table 1 lists the skin deformation amplitudes of the three levels (C2, C5, and C6) in the two neck flexion postures in three directions. Data show that the range of skin shift amplitude was 0–12.5 mm in the *X* direction, 0–13.5 mm in the *Y* direction, and 0–13.5 mm in the *Z* direction. Meanwhile, Table 2 lists the skin shift amplitudes of the three cervical levels at four postures in lateral bending. Results show that the range of skin shift amplitude was 0–21 mm in the *X* direction, 0.5–20.5 mm in the *Y* direction, and 0.5–16 mm in the *Z* direction. During flexion, the maximum skin displacements in both *X* and *Y* directions occurred at the C2 level, giving values of 8.5 and 7.2 mm, respectively. By contrast, the maximum skin displacement in the *Z* direction (up to 9 mm) occurred at C6. During lateral bending, the maximum skin displacements in the *X*, *Y*, and *Z* directions were noted on at the C2 level, with values of 14.5, 20.5 and 7.2 mm, respectively.

**Figure 3:**
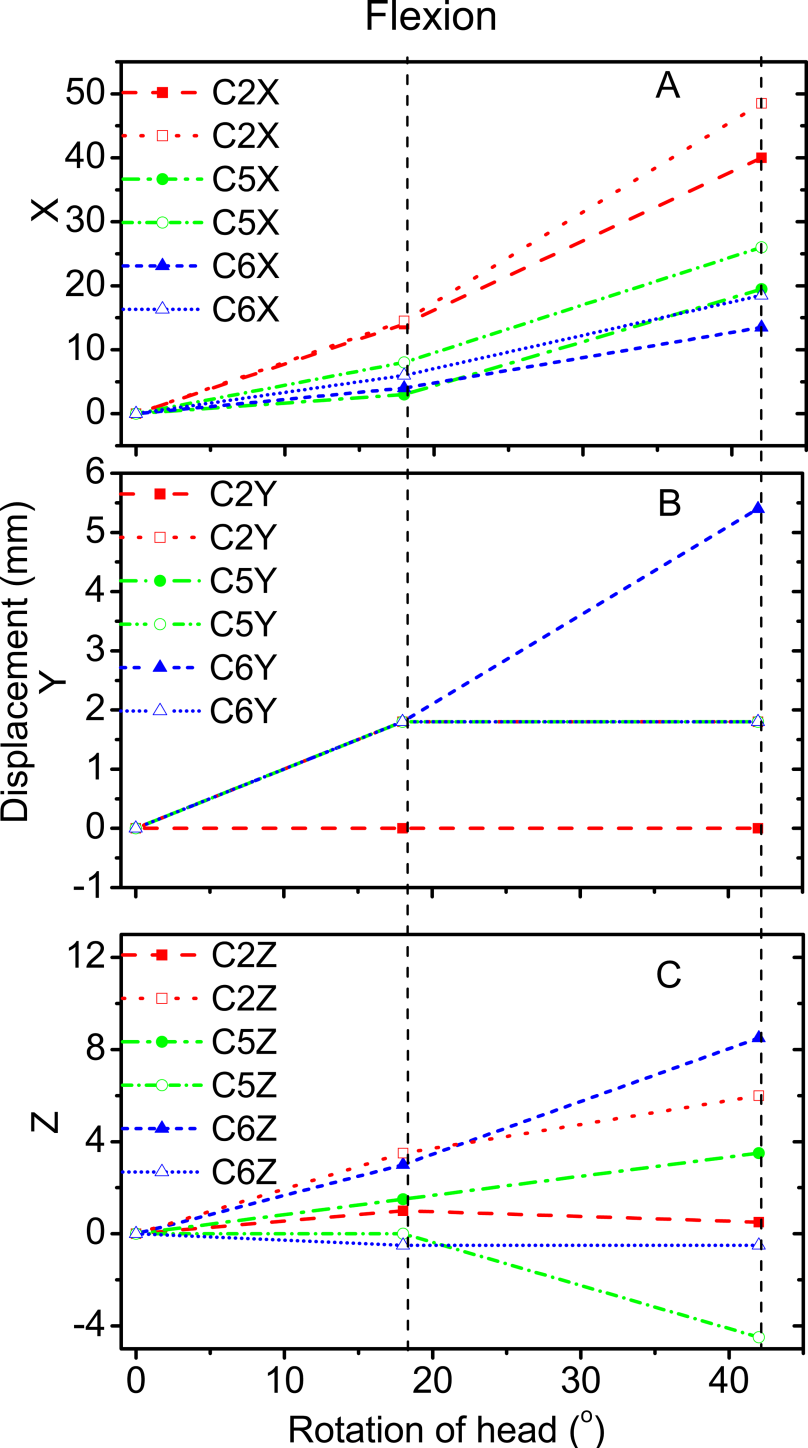
Motion displacement relative to the neutral positions of the vertebral landmarks and corresponding skin markers on the C2 (red), C5 (green), and C6 (blue) levels in *X* (A), *Y* (B), and *Z* (C) directions during flexion. C2-vertebral landmarks: dashed line; C2-skin markers: dotted line; C5-vertebral landmarks: dash dotted line; C5-skin makers: short dash dotted line; C6-vertebral landmarks: short dashed line; C6-skin markers: short dotted line.

**Figure 4:**
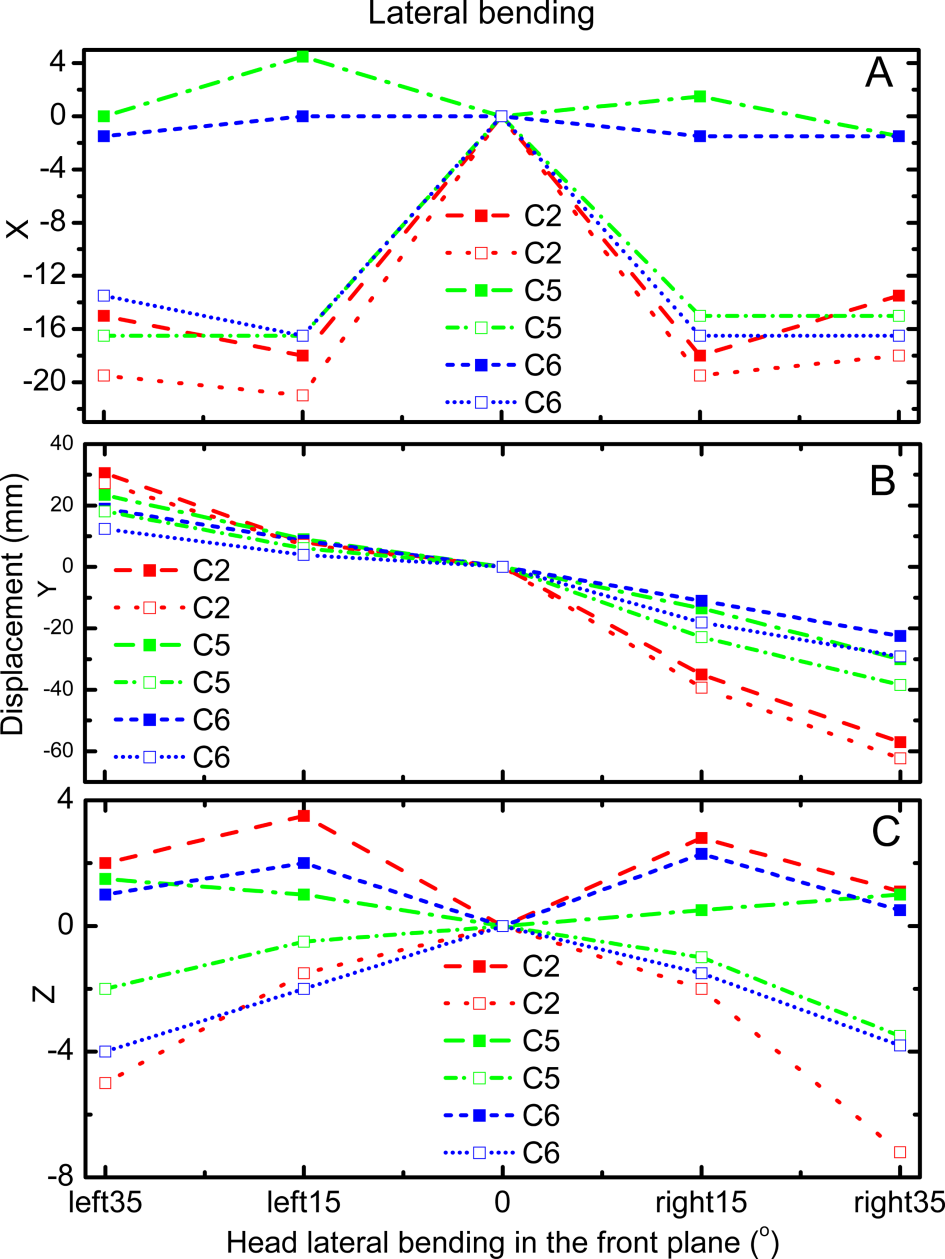
Motion displacement relative to the neutral positions of the vertebral landmarks and corresponding skin markers on the C2 (red), C5 (green), and C6 (blue) levels in *X* (A), *Y* (B), and *Z* (C) direction during lateral bending. C2-vertebral landmarks: dashed line; C2-skin markers: dotted line; C5-vertebral landmarks: dash dotted line; C5-skin makers: short dash dotted line; C6-vertebral landmarks: short dashed line; C6-skin markers: short dotted line.

Figures 3 and 4 reveal the motion of vertebral landmarks and the corresponding skin markers in flexion and lateral bending, respectively. The initial motion of vertebral landmarks and skin markers in the neutral position was eliminated to analyze the influence of skin deformation on the different cervical motion periods. The results demonstrated that the direction of movement of most of the skin markers in flexion could estimate the vertebral landmarks. In lateral bending, the motion directions of the skin markers of the upper cervical spine could estimate those of the corresponding vertebral landmarks both in the *X* and *Y* directions, as well as that of the lower cervical spine in the *Y* direction. Moreover, the motion directions of the skin markers of the lower cervical spine, as well as those of all cervical levels, were opposite those of the corresponding vertebral landmarks in the *X* and *Z* directions, respectively. These results imply that the skin movement characteristics differ between the upper and lower cervical levels, as embodied in the motion directional and amplitude differences. The discrepancies can be explained by the skin deformation mechanism caused by the complex cervical muscle deformation.

For the amplitude information during flexion, Fig. 3A shows that the skin displacement at the C5 level was larger than those of the other two levels in the *X* direction during the entire movement process. Meanwhile, Fig. 3B reveals that the maximum skin displacement in the *Y* direction occurred at the C2 level, followed by the C5, and then C6 levels. Fig. 3C shows that the maximum skin displacement in the *Z* direction occurred at the C6 level, then at the C5 and C2 levels.

During lateral bending, Fig. 4A shows that the maximum skin deformation in the *X* direction occurred at the C5 level and then the C6 and C2 levels. Meanwhile, the skin deformation presented symmetry in this direction. Fig. 4B displays that the maximum skin deformation in the *Y* direction occurred at the C6 level during left bending, whereas the maximum skin deformation occurred at the C5 level during right bending. Fig. 4C reveals that the maximum skin deformation in the *Z* direction occurred at the C2 level, followed by C6, and then C5 levels. The skin deformation was also symmetrical in this motion direction.

### Linear relationship analysis of the motions of the skin markers and vertebral landmarks

To quantify the relative movement relationship between skin markers and the corresponding vertebral landmarks, regression equations were analyzed. All data obtained above were included. Correlation coefficients were adopted as tools to determine the degree of correlation between two variables, particularly, the displacements of skin marker *T* andvertebral landmark *Y*. The linear correlations between these displacements were calculated. The simple linear regression equation was employed in this work (Mörl & Blickhan, 2006), as follows:

(2) \begin{eqnarray*}Y=bT+a.\end{eqnarray*}

The vertebral landmark displacement *Y* can be calculated using the superior skin marker displacement data *T*, estimated regression coefficient *b*, and intercept *a*. The change in postures led to a variation in marker and vertebral displacements. The Pearson’s correlation coefficients of the magnitudes of these displacements were calculated (0.07 < *R* < 0.97).

**Figure 5:**
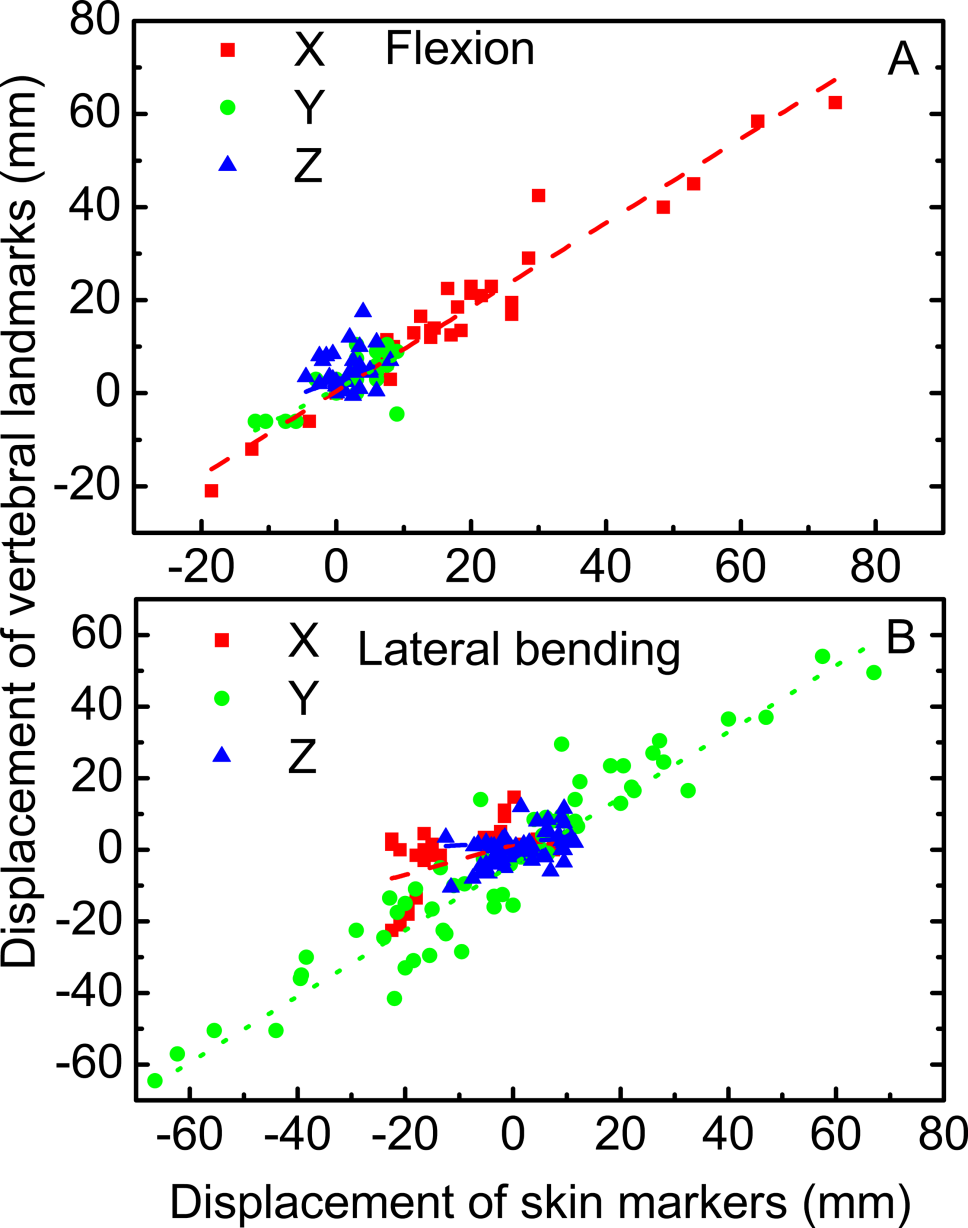
Linear relationship between vertebral landmarks and skin markers in the *X* (dashed lines in red), *Y* (dotted lines in green), and *Z* (dash dotted lines in blue) directions for the motion patterns of flexion (A) and lateral bending (B).

The linear relationships among the motions of the skin markers and vertebral landmarks in flexion and lateral bending are displayed in Fig. 5. For flexion, the correlation coefficients of the displacements of the vertebral landmarks and corresponding skin markers in the *X* direction were 0.95. This value was higher than those in the *Y* and *Z* directions at 0.77 and 0.30, respectively. For lateral bending, the correlation coefficient was 0.94 in the *Y* direction, corresponding to a primary direction. Meanwhile, the correlation coefficients were low in both the *X* and *Z* directions.

## Discussion

This work aimed to qualitatively and quantitatively investigate the cervical skin deformation at both upper and lower cervical levels. Three postures during flexion and five postures during lateral bending were involved. Furthermore, the linear correlation coefficients between the displacements of the skin markers and corresponding vertebral landmarks were investigated.

The motion direction of skin markers could correctly estimate that of the corresponding vertebral landmarks in flexion. In lateral bending, the motion directions of the skin markers could estimate the corresponding vertebral landmarks in the *Y* direction. By contrast, discrepancies were noted in the *X* and *Z* directions. The skin markers estimated the motion direction of the vertebral landmarks in the primary motion directions for the two motion patterns. However, in other directions outside of the primary motion plane, prudence was needed when skin markers were used to obtain the vertebral landmark motion. Moreover, the displacements of skin deformation observed in the study were slightly larger than those provided by Wu et al. (2007). With the aid of fluoroscopy, Wu et al. (2007) conducted measurements, in which the skin displacement might have been offset during the movement process. In the present study, no rectification was applied despite that the entire measurement was performed using the MRI technique. The natural motion patterns of cervical spine were performed although the images were obtained in static postures. The results showed that the amplitude of skin deformation increased with increasing cervical motion range. Larger ranges of cervical motion produced higher skin deformation amplitudes, which might be attributed to the greater participation of neck muscles. Moreover, in flexion, the correlation coefficients between the motions of skin markers and corresponding vertebral landmarks were high (*R* > 0.77) in the *X* and *Y* directions, but were poor in the *Z* direction. In lateral bending, the correlation coefficient was high (*R* > 0.94) in the *Y* direction. In the primary motion planes, the motion amplitude of vertebral landmarks can be estimated by the skin markers through linear regression equations both in flexion and lateral bending.

The displacement compensation approach has been used by Ryu, Choi & Chung (2009) to significantly reduce errors in keen kinematic variables by 25%–60%. Similarly, the displacement investigation of cervical skin deformation could provide a reference for the compensation of cervical angular information. Opinions on the biodiversity of different people prevail (Leardini et al., 2005). However, Gao & Zheng (2008) recently verified the inter-subject similarity of soft tissue deformation during level walking between two subjects. The sample size involved in the study potentially influenced the result. Such findings verified the prevailing opinion that skin deformation presented biovariability. However, the small sample size probably decreased the variability and increased the inter-subject similarity. Therefore, future studies involving larger sample sizes should be performed to completely verify the existing hypothesis on human cervical skin deformation. Objective subject information, such as age, sex, height, and gravity, may have affected the result. In the technical perspective, the limitation of this study may lie in the use of special self-made positional approach, the limited choice of landmarks, and the limited position of subjects. Further study needs to include completed vertebral landmarks, and more positions should be researched as well. However, additional information on study subjects should also be provided to accurately achieve a deeper understanding of the skin deformation mechanism. Moreover, with the widespread use of the cervical finite element model, the skin deformation results obtained in the study can be adopted to assist in the *in vivo* subject-specific validation of the finite element model of the cervical spine.

## Conclusions

A non-invasive MRI technique was implemented to investigate the skin deformation of several cervical segments in flexion and lateral bending. The amplitude and directional information on skin deformation in three directions was investigated. Results showed that the motion patterns of cervical skin deformation presented biovariability. This work offered reference for the further investigation of the cervical skin deformation mechanism.

## Additional Information and Declarations

### Data Availability

The following information was supplied regarding data availability: Figshare: https://figshare.com/articles/Soft_tissue_artifact_evaluation_of_the_cervical_spine_in_motion_patterns_of_flexion_and_lateral_bending_A_preliminary_study/2056386.
